# Comparing different metabolic indexes to predict type 2 diabetes mellitus in a five years follow-up cohort: The Baependi Heart Study

**DOI:** 10.1371/journal.pone.0267723

**Published:** 2022-06-03

**Authors:** Camila Maciel de Oliveira, Jessica Leticia Pavani, Chunyu Liu, Mercedes Balcells, Robson Capasso, Rafael de Oliveira Alvim, Carlos Alberto Mourão-Júnior, José Eduardo Krieger, Alexandre Costa Pereira

**Affiliations:** 1 Stanford University School of Medicine, Stanford, California, United States of America; 2 Laboratory of Genetics and Molecular Cardiology, Heart Institute (InCor), University of São Paulo Medical School, São Paulo, Brazil; 3 Department of Integrative Medicine, Federal University of Parana, Curitiba, Brazil; 4 Department of Statistics, Pontificia Universidad Católica de Chile, Santiago, Chile; 5 Framingham Heart Study, Framingham, Massachusetts, United States of America; 6 Department of Biostatistics, Boston University, Boston, Massachusetts, United States of America; 7 Institute for Medical Engineering and Science, Massachusetts Institute of Technology, Cambridge, Massachusetts, United States of America; 8 Bioengineering Department, Institut Quimic de Sarria, Ramon Llull Univ, Barcelona, Spain; 9 Department of Physiological Sciences, Federal University of Amazonas, Manaus, Brazil; 10 Department of Physiology, Federal University of Juiz de Fora, Juiz de Fora, Brazil; Faculdade de Medicina de São José do Rio Preto, BRAZIL

## Abstract

This study evaluates the association of anthropometric indexes and the incidence of type 2 diabetes mellitus (T2DM) after a 5-year follow-up. This analysis included 1091 middle-aged participants (57% women, mean age 47 ± 15 years) who were free of T2DM at baseline and attended two health examinations cycles [cycle 1 (2005–2006) and cycle 2 (2010–2013)]. As expected, the participants who developed T2DM after five years (3.8%) had the worst metabolic profile with higher hypertension, dyslipidemia, and obesity rates. Besides, using mixed-effects logistic regression and adjustment for sex, age, and glucose, we found that one unit increase in body adiposity index (BAI) was associated with an 8% increase in their risk of developing T2DM (odds ratio [OR] = 1.08 [95% CI, 1.02–1.14]) and visceral adiposity index (VAI) was associated with a risk increase of 11% (OR = 1.11 [95% CI, 1.00–1.22]). Moreover, a one-unit increase in the triglycerides-glucose index (TyG) was associated with more than four times the risk of developing T2DM (OR = 4.27 [95% CI, 1.01–17.97]). The interquartile range odds ratio for the continuous predictors showed that TyG had the best discriminating performance. However, when any of them were additionally adjusted for waist circumference (WC) or even body mass index (BMI), all adiposity indexes lost the effect in predicting T2DM. In conclusion, TyG had the most substantial predictive power among all three indexes. However, neither BAI, VAI, nor TyG were superior to WC or BMI for predicting the risk of developing T2DM in a middle-aged normoglycemic sample in this rural Brazilian population.

## Introduction

Type 2 diabetes mellitus (T2DM) is a problem approaching epidemic proportions globally. Data from the International Diabetes Federation indicated 463 million cases in 2019, expecting a 51% increase by 2045 [1]. Such a problem is even more severe, considering that an estimated 50% of cases remain undiagnosed [1]. Amid this global public health problem, Brazil ranks fifth in diabetic individuals in the world. About 16.8 million Brazilian adults are diagnosed with T2DM, which means one in nine has the disease. The causes for such a high number of cases have not been appropriately characterized, although many studies indicate a strong association with diet quality, lack of physical activity, and obesity. In Brazil, obesity is one of the most critical risk factors for various diseases [2], including T2DM.

Then the scientific community has been looking for clinical methods to prevent the T2DM onset. Previous studies have explored the association between some anthropometric indexes and T2DM, besides recognized obesity measurements such as waist circumference (WC) or body mass index (BMI). Developed by Bergman et al. [3], body adiposity index (BAI) is one of the most investigated proxies, showing to be associated with T2DM [4–6]. Alternatively, the visceral adiposity index (VAI) has gained prominence concerning the risk of metabolic diseases [7–10]. Another marker, which has proved essential in the study of T2DM, is the triglyceride-glucose index (TyG) [11–15].

Although numerous clinical studies have described the associations between these anthropometric indexes and the incidence of T2DM in European descent, these proxies have not been investigated in the Brazilian population. Ethnic differences may influence the discriminatory power of anthropometric markers in assessing the risk of T2DM [4, 16]. Therefore, in a longitudinal cohort study, our main interest was investigating the associations of BMI, WC, BAI, VAI, and TyG with the incidence of T2DM in a middle-aged normoglycemic sample in a Brazilian rural population. Also, our research aimed to identify the index that had the highest predictive power to predict T2DM.

## Methods

The Baependi Heart Study (BHS) started in 2005 in the southeast of Brazil [17]. A small town—Baependi—was chosen for the data collection process of this cohort. The individuals were randomly invited into the study (both sex and ages 18–102 years). Once recruited, the participants were examined every five years to obtain their demographic variables, medical history, blood samples, and clinical exams [17, 18]. At present, the cohort study consists of three health examination cycles. For this study we analyzed data from cycle 1 (2005–2006; n = 1712; 119 families) and cycle 2 (2010–2013; n = 3017; 127 families). In cycle 2, we have included participants that at that time were older than 17 years old and other relatives from original families that had not been enrolled previously in cycle 1. Also, some new families were included in cycle 2 (n = 9).

For this analysis, the final sample consisted of 1091 individuals, considering those participants that lost follow-up or died between cycles 1 and 2 (n = 422), missing data (n = 86), and also excluding any participant with the diagnosis of T2DM in the baseline (n = 113).

Based on the newly T2DM diagnosis in cycle 2, the eligible participants were divided into two groups: (i) Diabetes Free (those who remained without T2DM) and (ii) Incident Diabetes (those who developed T2DM). T2DM was defined by the presence of fasting plasma glucose ≥126 mg/dL or the use of any hypoglycemic drugs [19].

Initially, all the participants answered a questionnaire [17, 18] in which they stated whether they were users of any medication to control blood glucose levels, blood pressure, and lipids. Additionally, all participants were referred for blood screening [18] regardless of answers to these questions. Besides that, blood pressure and anthropometric parameters (weight, height, and waist circumference) were measured [18].

Increased waist circumference (WC) was defined as >88 cm for women and >102 cm for men [20]. Hypertension, when mean SBP ≥ 140 mmHg or DBP ≥ 90 mmHg or antihypertensive drug use [20]. Dyslipidemia treatment was defined when at least one class of lipid-lowering drugs was used [20].

The remaining anthropometric indexes—BAI, VAI, and TyG—were calculated using the following Equations [3, 7, 12]:

$$\text{BAI}=\left(\text{Hip}\;\text{Circumference}\;\left(\text{cm}\right)/{\text{Height}}^{1.5}\;\left(\text{m}\right)-18\right)$$

VAI

Males={waistcircumference/[39.68+(1.88*bodymassindex)]}*(tryglicerides/1.03)*(1.31/HDL-c)

Females={waistcircumference/[39.58+(1.89*bodymassindex)]}*(tryglicerides/0.81)*(1.52/HDL-c)

TyG=ln[(Triglycerides*Glucose)]/2

Continuous variables were normally distributed and were expressed as the mean ± SD. Categorical variables were expressed as percentages. Both were used for describing the clinical characteristics in cycles 1 and 2. We performed analysis using the mixed-effects logistic regression to evaluate the association between some indexes—BAI, VAI, and TyG—and the incidence of T2DM. Mixed-effects logistic regression was also used to assess the risk for developing T2DM. The regression model was carried out with BAI, VAI, or TyG as the main predictor variables (adjusted for sex and age, Model 1) or also for glucose (Model 2) and another adiposity index (Model 3). Considering the kinship relations among individuals, we also took into account family as a random effect. Results from the mixed-effects logistic regression were also reported as odds ratios per interquartile range (IQR-OR) with their 95% confidence intervals [21]. In the logistic regression model, we chose to calculate the odds ratio (OR) relative to the interquartile range (IQR) because we are comparing indices that are not measured on the same scale. In this way, when we use quartiles (in this case, the 25th and 75th percentiles), we standardize the measures, making them commensurate with each other. This advantage allows comparisons to be made, which is our primary objective. Conversely, in doing so, the clinical interpretation of the regression model is somewhat impaired, as what the model will tell us is how much the risk increases for each unit that the IQR increases. Although the clinical application of this information is not straightforward (since we do not routinely use the IQR in health practice), this artifice allowed us to compare the indices, as the quartiles will always be the values that divide the values into four classes, regardless of the scale of measure. The R Studio software (Version 1.3.1093) was used for analyses [22] and the level of significance was set at 5%.

The Baependi Heart Study has an ethics approval (Hospital das Clínicas, SDC 3485/10/074, University of São Paulo). Each participant consented to be part of this cohort, previously the data collection, by assigning a written informed consent. The data were analyzed anonymously.

## Results

After a 5-year follow-up, 3.8% of the normoglycemic group at baseline developed T2DM (57% women, mean age 57 ± 12 years) (Table 1). The worse metabolic profile was shown in the Diabetes Incident group, which was older and had a higher proportion of obesity, increased WC, hypertension, and lipid-lowering drugs. Mean levels of triglycerides, BAI, VAI, and TyG, were also higher in the group that developed T2DM.

**Table 1:** Clinical characteristics at baseline (Cycle 1) and 5-year follow-up (Cycle 2) in the normoglycemic sample of the Baependi Heart Study.

Variable	Cycle 1	Cycle 2	
		Diabetes-free group	Incident Diabetes group
n	1091	1049 (96.2%)	42 (3.8%)
Age, years	42 ± 15	47 ± 15	57 ± 12**
Sex (% men)	43	43	33
Hypertension (%)	31	38	70**
Increased WC (%)	27	39	61*
Obesity	12	20	39*
Dyslipidemia treatment (%)	3	7	22**
Current smoke (%)	14	12	12
SBP, mmHg	125 ± 19	126 ± 16	131 ± 16
DBP, mmHg	79 ± 11	77 ± 10	79 ± 11
BMI, kg/m2	24 ± 5	26 ± 5	29 ± 5**
WC, cm	87 ± 12	91 ± 11	100 ± 11**
Fasting glucose, mg/dL	87 ± 17	90 ± 10	141 ± 51**
Total cholesterol, mg/dL	179 ± 48	198 ± 40	194 ± 43
HDL-cholesterol, mg/dL	57 ± 16	48 ± 13	48 ± 11
LDL-cholesterol, mg/dL	97 ± 43	125 ± 34	114 ± 36
Triglycerides, mg/dL	129 ± 70	128 ± 65	162 ± 88*
TyG	4.60 ± 0.26	4.62 ± 0.24	4.93 ± 0.35**
BAI	28.56 ± 6.54	29.26 ± 6.10	32.30 ± 6.27*
VAI	4.04 ± 2.70	4.87 ± 3.22	6.58 ± 4.68*

SBP, systolic blood pressure; DBP, diastolic blood pressure; BMI, body mass index; WC, waist circumference; TyG, triglycerides-glucose index; BAI, body adiposity index; VAI, visceral adiposity index. Hypertension: systolic blood pressure ≥140 mmHg, diastolic blood pressure ≥90 mmHg, and/or anti-hypertensive drug use. Dyslipidemia treatment: percentage of individuals who used at least one class of lipid-lowering drug. Increased WC, > 88 cm for women and >102 cm for men. Continuous data are expressed as the mean ± standard deviation and categorical data are expressed as percentage.

*(p<0.05)

** (p<0.001) Incident Diabetes versus Diabetes Free in Cycle 2

Health examination was conducted at Cycle 1 (2005 to 2006) and Cycle 2 (2010 to 2013).

Baependi Heart Study, Brazil (2005–2013)

The best combination to predict the incidence of T2DM, according to the area under the curve (AUC) goodness of fit assessment in binary logistic regression, was sex, age, BAI, VAI, and TyG in the same model (0.891) or sex, age, and WC together (0.891). Then, a complete model (sex, age, BAI, VAI, and TyG) was carried out to verify the significance of those indexes (Table 2). We identified that BAI was significantly different between participants with T2DM and free of the disease (p-value < 0.05), even adjusted for age, sex, and glucose. However, the model did not suffice when BAI was additionally adjusted for WC or BMI.

**Table 2:** AUC used to evaluate the goodness of fit in logistic regression in different models.

	Covariates	Age and Sex	Age, Sex, FBG	Sex, Age, FBG, BMI	Sex, Age, FBG, WC
Predictors	VAI	1.00 (0.86–1.18)	1.04 (0.88–1.24)	1.04 (0.87–1.25)	1.02 (0.85–1.23)
	BAI	1.07 (1.01–1.14)*	1.08 (1.01–1.14)*	0.95 (0.85–1.06)	0.99 (0.90–1.08)
	TyG	4.00 (0.52–30.70)	2.13 (0.19–23.59)	1.38 (0.11–17.08)	1.77(0.14–21.96)

Dependent variable is T2DM, Independent variable is VAI, BAI or TyG. Covariates are age, sex, FBG, BMI and WC.

BAI, body adiposity index; TyG, triglycerides-glucose index; VAI, visceral adiposity index; WC, waist circumference; BMI, body mass index; FBG, fasting blood glucose

The results are expressed by OR, odds ratio; CI, confident interval;

*(p<0.05)

Health examination was conducted at Cycle 1 (2005 to 2006) and Cycle 2 (2010 to 2013).

Baependi Heart Study, Brazil (2005–2013).

Then, some other models for each index were carried out. In Table 3, Model 1 accounts for age and sex; Model 2, age, sex, and glucose; Model 3, age, sex, glucose, or another obesity measurement (BAI, VAI, or TyG). Using mixed-effects logistic regression, we observed that BAI, VAI, and TyG were significantly different between participants with T2DM and free of the disease when adjusted for sex and age (Model 1). In model 2, we could show that the elevation of each unit represented an increased risk of 8% for developing diabetes when BAI was analyzed. (odds ratio [OR] = 1.08 [95% CI, 1.02–1.14]; p<0.05). Besides, when analyzing TyG in model 2, we noticed that one unit increase in TyG was associated with four times the risk of developing T2DM (OR = 4.27 [95% CI, 1.01–17.97]; p<0.05), even adjusted for glucose. In model 3, none of the indexes—BAI, VAI, and TyG—could suffice when adjusted for WC or BMI. Oppositely, when we analyzed WC or BMI, both were significant in all models.

**Table 3:** Odds ratio in three different models of binary logistic regression (dependent variable is T2DM).

		Model 1	Model 2	Model 3				
	Covariates	Age and Sex	Age, Sex, and Glucose	Age, Sex, Glucose and obesity indexes (VAI, BAI, TyG, WC and BMI)				
				VAI	BAI	TyG	WC	BMI
Predictors	WC	**1.06 (1.03–1.09)** **	**1.06 (1.03–1.09)** **	**1.06 (1.02–1.09)** *	**1.06(1.02–1.11)** *	**1.06(1.04–1.08)** **	-	1.04 (0.98–1.10)
	BMI	**1.13 (1.06–1.20)** **	**1.12 (1.06–1.20)** **	**1.12 (1.05–1.20)** *	**1.17(1.04–1.32)** *	**1.12 (1.05–1.20)** **	1.04 (0.91–1.19)	-
	VAI	**1.11 (1.0–1.22)** *	1.11 (1.0–1.22)	-	1.09(0.98–1.20)	1.05 (0.88–1.25)	1.06 (0.95–1.18)	1.07 (0.96–1.18)
	BAI	**1.08 (1.02–1.14)** *	**1.08 (1.02–1.14)** *	**1.08 (1.02–1.14)** *	-	**1.07 (1.01–1.14)** *	0.99 (0.91–1.08)	0.96(0.86–1.16)
	TyG	**4.55 (1.21–17.16)** *	**4.27 (1.01–17.97)** *	2.4 (0.21–27.37)	3.22(0.73–14.21)	*-*	2.25(0.50–10.09)	2.23 (0.49–10.04)

Model 1, accounting for Age and Sex; Model 2, accounting for Age, Sex and Fasting Blood Glucose; Model 3, accounting for Age, Sex, Fasting Blood Glucose and each measurement of obesity.

*(p<0.05)

** (p<0.001)

To compare the predictive power of these indexes, we calculated IQR-OR and their respective confidence intervals (Table 4). Such an approach allowed us to reach predictors measured on different scales. After performed log-transformation followed by conversion to the exponential form, IQR-OR was 1.35 (95% CI, 0.30–6.04; p>0.05) for TyG and 1.16 for VAI (95% IC, 1.04–1.30; p>0.05), indicating that an IQR was associated with an increased risk for developing T2DM of 35% and 16%, respectively.

**Table 4:** Variables associated with diabetes mellitus in the Baependi Heart Study.

Variable	Beta	OR [95% CI]	IQR-OR [95% CI]	*p-value*
Sex	-1.24	0.29 [0.11, 0.79]	0.54 [0.20, 1.48]	0.23
Age	0.04	1.04 [0.73, 1.48]	2.25 [2.20, 2.30]	**0.003**
WC	0.06	1.06 [0.65, 1.74]	2.57 [2.47, 2.68]	**0.006**
Glucose	0.02	1.02 [0.72, 1.43]	1.44 [1.41, 1.47]	0.14
BAI	-0.01	0.99 [0.56, 1.74]	0.90 [0.83, 0.98]	0.78
Sex	-0.83	0.43 [0.21, 0.88]	0.66 [0.33, 1.34]	0.25
Age	0.03	1.03 [0.72, 1.48]	2.12 [2.07, 2.17]	**0.01**
WC	0.06	1.06 [0.75, 1.50]	2.50 [2.43, 2.58]	**0.0002**
Glucose	0.01	1.01 [0.70, 1.45]	1.13 [1.10, 1.15]	0.67
TyG	0.81	2.25 [1.53, 3.31]	1.35 [0.30, 6.04]	0.28
Sex	-0.89	0.41 [0.20, 0.85]	0.64 [0.31, 1.33]	0.23
Age	0.03	1.03 [0.72, 1.49]	2.18 [2.13, 2.23]	**0.01**
WC	0.05	1.06 [0.74, 1.50]	2.38 [2.31, 2.45]	**0.0003**
Glucose	0.01	1.01 [0.79, 1.43]	1.36 [1.34, 1.39]	0.22
VAI	0.06	1.06 [0.79, 1.42]	1.16 [1.04, 1.30]	0.32

Logistic regression was utilized to evaluate associations between diabetes mellitus (outcome) with predictor variables in a multivariate model. WC, waist circumference; BAI, body adiposity index; TyG, triglycerides-glucose index; VAI, visceral adiposity index; OR, odds ratio; CI, confident interval; IQR-OR, odds ratio for interquartile range.

Diabetes mellitus: diagnosis was established in patients with fasting glucose equal to or greater than 126 mg/dL, or in patients who were under the use of anti-diabetic drugs.

Predictive variable: BAI, TyG, and VAI, respectively.

Control variables: sex, age, glucose levels, and WC.

Health examination was conducted at Cycle 1 (2005 to 2006) and Cycle 2 (2010 to 2013).

Baependi Heart Study, Brazil (2005–2013).

## Discussion

This study analyzed the association between three main adiposity indexes—BAI, TyG, and VAI—and T2DM in a Brazilian population. In particular, the Baependi Heart Study included a rural Brazilian sample. To the best of our knowledge, the ability of these three clinical markers to predict T2DM in a Brazilian study with a large sample size has not been previously performed. Overall, our findings corroborated numerous studies which have demonstrated the importance of the anthropometric indexes in different populations, highlighting BAI, TyG, and VAI as quite useful. Such indexes can be used as good predictors for T2DM in Asian [8–11, 23], American [3], European [5], and Latin American populations [4, 6, 14, 16]. In Brazil, both Flor and Campos [24] and Freitas and Moraes [25] showed a strong association between T2DM and obesity, considering different Brazilian samples. Moreover, in the same direction as the findings of Bergman et al. [3] and López et al. [5], Oliveira et al. [6] had already pointed out the role of the body adiposity index to predict T2DM risk in the Baependi population.

In agreement with some reports [6, 14], we described a significant association between the three index variables—BAI, TyG, and VAI—and the incidence of T2DM when adjusted for age and sex. As shown before, we identified that one added unit in each index implies a significant increase in the risk of developing T2DM. However, a straight comparison among their respective OR values is not indicated due to the different scales of variables. Hence, we calculated the IQR-OR, which is comparable in magnitude for all the risk factors. The IQR describes the distance between the 25th and 75th percentiles. Then, the advantage of using IQR as a scaling factor is that, unlike the standard deviation, it reflects the values of the predictor that are relatively well-represented in the sample [21]. The resulting regression coefficient compares a value in the middle of the upper half of the predictor distribution to a value in the middle of the lower half of the distribution. To date, we know of only one study that compared the predictive power of TyG, BMI, and WC for prediabetes or diabetes. This study demonstrated that the TyG was best able to identify prediabetes in either sex, suggesting that compared to the other parameters (BMI and WC), the TyG has the best discriminative power to predict prediabetes in the whole population [16]. Using statistical modeling and IQR measure (Similar to Ramírez-Vélez et al. [16]), our study showed that TyG was not superior to WC or BMI for predicting the risk of developing T2DM.

The superiority of TyG over BAI and VAI is likely to be clinically explained by the fact that it is associated with insulin resistance, which is known to be a determining factor in the etiopathogenesis and pathophysiology of T2DM [15, 26]. In epidemiological and clinical studies, the advantage of the TyG index is less costly than other insulin markers and also due to glucose and triglycerides are biochemical tests routinely performed in the primary care setting [15].

Regarding clinical applicability, our objective was to identify the highest precision and accuracy index that allows physicians to make clinical decisions and apply preventive measures in individuals with increased risk for T2DM. In addition, the three indexes studied are easy to obtain from clinical and laboratory data that are generally requested in the routine of follow-up consultations. Thus, we suggest the TyG index as a complementary marker for assessing T2DM in clinical practice and future epidemiological studies among Brazilian adults.

Conversely, TyG (as well as the other adiposity indexes) loses its predictive value when the statistical models are adjusted for conventional measures of body fat (BMI and WC). If this assumption becomes reproducible in new similar studies, it will be plausible to surmise that these easily obtainable measures in clinical practice remain valuable. From a clinical and pathophysiological point of view, this makes sense since it corroborates the thesis that obesity is probably the major risk factor for the development of T2DM. Indeed, this fact was expected in our study since we evaluated patients over a 5-year interval, using BMI and WC as covariates, in as much as the expectation is that weight gain occurs over time.

Furthermore, we are aware that our findings are particular to a rural population and that other studies are likely to have different risk factors. For instance, it is known that the adult’s ethnicity [4, 16] can influence the anthropometric indicators associated with glycemic status. Such influence could impact their association with T2DM. Further investigations are warranted to provide reference values applicable to different ethnic populations.

This study has a few limitations. First, all participants live in Baependi, a small town with great rural activity in South-eastern Brazil. Therefore, these results may not be easily generalized to represent the overall Brazilian population. Second, this work was based on an observational study where the participant makes a single visit per cycle. So, analogous to other studies, T2DM was defined based on a single fasting blood glucose measurement or a report of hypoglycemic drug use.

To sum up, our results show that TyG has a more significant potential than VAI and BAI as a predictor of T2DM. However, measures easily obtained in clinical practice (BMI and WC) seem to maintain their hegemony.

## Abbreviations

BAI: body adiposity index
BMI: body mass index
CI: Confidence interval
DBP: diastolic blood pressure
FPG: fasting plasma glucose
IQR: interquartile range odds ratio
OR: odds ratio
SBP: systolic blood pressure
SD: standard deviation
T2DM: type 2 diabetes mellitus
TyG: triglycerides-glucose index
VAI: visceral adiposity index
WC: waist circumference
